# Individual Remedy in Epilepsy

**Published:** 1862-09

**Authors:** 


### INDIVIDUAL REMEDIES IN EPILEPSY.

Dr. Anstie read a paper before the Medical Society of London (March 24, 1862,) in which he gave the results of his own experience in the use of certain remedies in epilepsy. These were of two kinds—tonics and sedatives: of the former he had made careful experiments with cod liver oil, quinine, and phosphorus, and also to a certain extent with iron; but of the effects of this last remedy he had not kept a tabulated record. Cod-liver oil was employed *alone* in twelve cases among the out-patients of Westminster Hospital and Chelsea Dispensary: the disease was in every instance of the simple or "idiopathic" kind; and the following results were obtained: Three complete failures (patients aged 14, 26, and 32, respectively; in all of them the disease was of long standing); one case in which the patient, a man, aged 44, derived no relief as far as regarded the fits, but his mental condition was improved; two cases (aged 10 and 12) which were improved, the fits being lessened in frequency, but the patients disappeared from the author's supervision while still by no means cured; and, finally, six cases, all still under observation, in which the fits have ceased entirely, and, so far as can be seen, the disease has been cured. Out of these six cases, two were sufficiently grave to put the efficacy of the remedy to a severe test; one of them was that of a girl, aged 17, epileptic from infancy, and in whom the fits had for some time past been happening two or three times every day; the effect upon the facial aspect and upon the intelligence of the patient had been very serious. Cod liver oil was employed persistently for three months with the effect of inducing an entire cessation of the fits, and a most marked improvement in the intellectual and sensorial functions. The other case was that of a boy, aged 13, in whom the disease, which was hereditary, had come on six months previously. The fits were not very frequent, but were very severe; the memory was a good deal affected, and the shape of the head and the aspect of the face were far from encouraging. Nevertheless, a complete cessation of the fits soon followed the use of cod-liver oil. The remedy was persisted in for six months on account of occasional threatening symptoms; but even these have quite disappeared. The mental condition is much improved, and the boy may be pronounced cured, as far as we dare use that term at all. Quinine had been used singly in six cases: two of them had entirely resisted its influence (both these patients were women), a third had been very much benefited, the fits being much reduced in frequency, and an unpleasant numbness in the leg having disappeared. In the other three the improvement apparently amounted to cure. One of these cases, in which a very marked and peculiar aura was present, was minutely analyzed, and the influence of quinine in stopping the aura, and altogether averting the paroxysms, was plainly shown. Doubtless local measures might have done much in this case, but they were intentionally and successfully dispensed with. All the cases treated by the author with quinine had been distinguished by the presence of some persistent local pain or numbness. Phosphorus had been tried by the author in only two cases of epilepsy, at the suggestion of his friend and colleague, Dr. Radcliffe, who has recommended its use in his book, as also that of cod-liver oil. The cases were inveterate ones, in which cod-liver oil had failed to produce any effect. The phosphorus was given in doses of five to ten drops of the phosphoretted oil of the Prussian Pharmacopœia, three times a day. The fits were not lessened in frequency, but the general condition of the patients was much improved, and the miserable sense of nervous depression greatly relieved. Phosphorus ought to be extensively tried in epilepsy and in other nervous affections. With regard to iron, the author not having kept tabulated reports of its effects, was only able to confirm, in a general way, the popular belief in its great utility. He was in the habit of limiting its employment to cases distinguished by anæmia; and he related one or two instances of its usefulness when given in such cases. The author remarked that all these four tonics, cod-liver oil, steel, quinine, and phosphorus, were distinguished by the fact that they acted as *foods*, either to the nervous system, or the blood that nourishes that system. Looking at the success obtained by himself, and by other observers of larger experience, and also to the fact that the whole group of chronic convulsive diseases, of which epilepsy may be said to form the centre, are curable, if at all so, in nine cases out of ten by a nutrient-tonic regimen—the author urged that there was the greatest reason for extended experiments with remedies of this class, experiments which should be patiently persisted in for months together, no other medicines being used, except aperients when necessary. The sedatives of which the author had made particular trial were opium, hyoscyamus, belladonna, and sulphate of aniline. With regard to belladonna, he must state that he had not thought it right to push it to the extent of producing wide dilatation of the pupil for a long time together. He had administered it in doses of one-sixth grain of the extract twice or three times a day, a quantity which is usually sufficient to produce the minor effects of the drug, namely, relief of neuralgic pain, and resolution of muscular spasms; and in these doses the results were very equivocal, and by no means encouraging. With regard to the other three sedatives, the author regarded it as proved by his own experience, that in a considerable number of instances they possess the power of delaying the fit, or mitigating its severity; and for this purpose he was at present inclined to give the preference to the sulphate of aniline. This remedy he had tried repeatedly upon six epileptic patients, and also in many other cases of chronic convulsive disease. It was a most serious mistake to administer sulphate of aniline, or indeed any other sedative, in large doses, with a view to arrest or diminish convulsive muscular action. In the only two cases of epilepsy in which the author had pushed it to the extent of large doses, on account of the failure of small quantities, a serious aggravation of the fits occurred. In doses of one grain three times a day, with an additional grain to be taken immediately on the occurrence of any *prodromata* of a fit, sulphate of aniline seemed to materially benefit four patients to the extent of delaying or mitigating the paroxysm, and in three separate instances the fit seemed to have been altogether averted for a considerable time.

In conclusion, the author desired to state his conviction that future experiments with sedatives should be limited to the use of small doses only, and that they should be employed chiefly in the slighter cases of epilepsy which are not of long standing, or in those in which tonics and nutrition had already done much, but had not quite effected a cure. In short, wherever we could reasonably hope to effect good by the mere fact of breaking through the vicious habit, so to speak, of convulsion; then we might well try experiments with sedatives, and the results obtained by each observer should be numerically noted, and published.—*Med. Times and Gaz.*

